# Sensitization to Food and Aero-Allergens in Children with Coeliac Disease Assessed with the Use of a Multiplex Molecular Diagnostic Technique

**DOI:** 10.3390/jcm13102992

**Published:** 2024-05-19

**Authors:** Izabela Knyziak-Mędrzycka, Bożena Cukrowska, Wojciech Nazar, Joanna Beata Bierła, Kamil Janeczek, Paulina Krawiec, Weronika Gromek, Mariusz Wysokiński, Ewa Konopka, Ilona Trojanowska, Sylwia Smolińska, Emilia Majsiak

**Affiliations:** 1 Allergology Clinic, The Children’s Memorial Health Institute, Aleja Dzieci Polskich 20, 04-730 Warsaw, Poland; 2 Department of Pathomorphology, The Children’s Memorial Health Institute, Aleja Dzieci Polskich 20, 04-730 Warsaw, Poland; 3 Faculty of Medicine, Medical University of Gdansk, Marii Sklodowskiej-Curie 3a, 80-210 Gdansk, Poland; wojciech.nazar@gumed.edu.pl; 4 Department of Clinical Biochemistry, The Children’s Memorial Health Institute, Aleja Dzieci Polskich 20, 04-730 Warsaw, Poland; j.bierla@ipczd.pl (J.B.B.);; 5 Department of Pulmonary Diseases and Children Rheumatology, Medical University of Lublin, Profesora Antoniego Gębali Street, 20-093 Lublin, Poland; kamil.janeczek@umlub.pl; 6 Department of Pediatrics and Gastroenterology, Medical University of Lublin, Profesora Antoniego Gębali Street, 20-093 Lublin, Poland; paulina.krawiec@umlub.pl; 7 Polish-Ukrainian Foundation of Medicine Development, Nałęczowska 14, 20-701 Lublin, Poland; weronikaa.gromek@gmail.com; 8 Department of Basic Nursing, Faculty of Health Sciences, Medical University, Staszica 4/6, 20-081 Lublin, Poland; mariusz.wysokinski@umlub.pl; 9 Department of Clinical Immunology, Wroclaw Medical University, Parkowa 34, 51-616 Wroclaw, Poland; sylwia.smolinska@umw.edu.pl; 10 Department of Health Promotion, Faculty Health of Sciences, Medical University of Lublin, Staszica 4/6, 20-081 Lublin, Poland

**Keywords:** coeliac disease, allergy, sensitization, specific immunoglobulin E, multiplex testing, molecular allergy diagnosis

## Abstract

**(1) Background**. Coeliac disease (CD) often co-occurs with autoimmune conditions or genetic syndromes, but there are few studies on the co-existence of CD and immunoglobulin E (IgE)-mediated allergies. The purpose of this study was to assess sensitization to food and aero-allergens in pediatric patients with CD. **(2) Methods**. A multiplex ALEX^®^2 test was used to determine specific IgEs (sIgEs). **(3) Results**. The study included 108 children newly diagnosed with CD. Allergen extract- and/or allergen molecule-sIgEs were detected in 49.1% of children. Most children (41.5%) were sensitized to both inhalant and food allergens. The three most common aero-allergens (timothy pollen, ryegrass, silver birch) were molecules Phl p 1, Lol p 1, and Bet v 1. The most common food allergens (hazelnut, apple, and peanut) were Cor a 1, Mal d 1, and Ara h 8 molecules of the PR-10 subfamily. Patients were not sensitized to cereal allergens containing gluten. Spearman’s rank correlation analysis of sensitized patients showed a significant positive relationship (*r* = 0.31) between the patients’ age and the occurrence of positive sIgEs (≥0.3 kU_A_/L) for inhalant allergen molecules (*p* = 0.045). In sensitized patients, mainly symptoms of inhalant allergy were observed, such as hay fever, conjunctivitis, and bronchial asthma. **(4) Conclusions**. The current study indicates the co-occurrence of IgE sensitization to food and inhalant allergens in children with CD. The study highlights the need to take a closer look at the diagnosis of IgE-mediated allergy in patients with CD, which may help in their care and lead to a better understanding of the relationship between CD and IgE-mediated allergy.

## 1. Introduction

Coeliac disease (CD), which is one of the most common gluten-induced food intolerances, affects people of all ages and manifests with both gastrointestinal and extra-gastrointestinal symptoms [1]. The gastrointestinal symptoms of CD include primarily abdominal pain, bloating, diarrhea, or constipation. The extra-gastrointestinal symptoms may include chronic fatigue, headaches, anemia, and elevated liver enzyme levels. Although CD has long been known to co-occur with autoimmune conditions (such as type 1 diabetes mellitus, autoimmune thyroid, or liver diseases) or genetic syndromes (such as Down syndrome, Turner syndrome, and Williams syndrome) [2], there have been few studies on the co-existence of CD and immunoglobulin E (IgE)-mediated allergies [3]. In CD, the sole factor that induces autoimmunity are immunoreactive gluten peptides, whereas in allergies, the causative factors may be either inhalant or food allergens and other (whose route of exposure is other than via inhalation or ingestion, for instance, via insect stings or via direct dermal contact). However, both types of conditions are associated with a dysfunctional immune system at large and regulatory T-cells specifically. Normally, regulatory T-cells help maintain immune tolerance and prevent both autoimmune and allergic conditions [4,5,6]. The pathophysiology of CD involves activation of helper T-cells 1 (Th1), followed by secretion of pro-inflammatory cytokines, and production of auto-antibodies directed against the enzyme tissue transglutaminase (tTG) [7]. The pathophysiology of IgE-mediated allergies involves activation of helper T-cells 2 (Th2) with a different cytokine profile than that in autoimmunity, which leads to overproduction of allergen-specific IgEs (sIgEs), and activation of sensitization process with the release of allergic reaction mediators by basophils and mast cells [6]. For these reasons, CD and IgE-mediated allergy are believed to be mutually exclusive, and the nature of the condition (whether autoimmune or allergic) is determined by the imbalance in Th1 and Th2 activation.

Despite the seemingly mutually exclusive pathogeneses of CD and IgE-mediated allergy, there have been studies indicating the co-existence of both conditions. A systematic review of studies by our research group [3] revealed a handful of original articles [8,9,10,11] in which authors reported that patients with CD may develop an allergy that manifests in a similar way to that in non-CD subjects, i.e., with atopic dermatitis, vomiting, urticaria, angioedema, anaphylactic shock, etc. In those studies, patients with CD were most often allergic to wheat, which may be due to the fact that this allergen was the one most frequently studied.

Co-occurrence of IgE-mediated allergy or sensitization in patients with CD has not been fully elucidated [6]. There is a need for studies on the comorbidity of CD and IgE-mediated allergy, not the least due to the fact that symptoms of CD often persist, despite the patients’ restrictive gluten-free diet—the only effective treatment [12]. This phenomenon may be due to an undiagnosed allergy to food and inhalant allergens. Therefore, the purpose of this study was to comprehensively assess sensitization to food and aero-allergen extracts and molecules with a multiplex ALEX^®^2 test in pediatric patients with CD.

## 2. Materials and Methods

### 2.1. Subjects and Study Design

The study included children newly diagnosed with CD at the Department of Gastroenterology, Hepatology, Nutritional Disorders, and Pediatrics at the Children’s Memorial Health Institute in Warsaw, Poland, and the gastroenterology outpatient clinic at the University Children’s Hospital in Lublin, Poland. CD diagnosis was established according to European Society for Paediatric Gastroenterology Hepatology and Nutrition (ESPGHAN) guidelines [13]. Tests for anti-tTG immunoglobulin A (IgA) and total IgA levels (Thermo Scientific Phadia GmbH, Freiburg, Germany) were conducted in all children. In children with IgA deficit, anti-tTG-IgG or anti-deaminated gluten peptide (DGP)-IgG levels were measured. Patients with high anti-tTG-IgA levels (>100 AU/mL, i.e., 10 times higher than the upper limit of normal) and positive anti-endomysial antibody tests (Euroimmun, Lübeck, Germany) (from a different blood sample) were diagnosed with CD without intestinal biopsy and a histopathological examination of small-intestinal specimens. In other patients, biopsies were taken during endoscopy (at least 1 specimen from the duodenal bulb and 4 specimens from the distal part of the duodenum) and histological analyses were performed by two independent pathologists using a modified Marsh–Oberhuber classification [14]. CD was diagnosed in children with positive antibodies (≥10 AU/mL) and histological changes described at least as Marsh 2.

Serum sensitization profiles were performed with the use of a multiplex molecular diagnostic technique (ALEX^®^2 test, Macro Array Diagnostics GmbH, Vienna, Austria) in the same sera that were used for CD diagnosis (anti-tTG test).

The study was approved by the local Ethics Committee of Medical University in Lublin, Poland (No. KE-0254/222/10/2023 of 23 October 2023). The approval of the Ethics Committee concerned the inclusion in the analysis of research results obtained as part of an earlier statutory project of the Children’s Memorial Health Institute, Warsaw, Poland (project number S168/2018, principal investigator B. Cukrowska). The project S168/2018 previously obtained consent from the Bioethics Committee at the Children’s Memorial Health Institute Warsaw, Poland (approval No. 50/KBE/2018 of 21 November 2018). Written informed consent was obtained from each patient’s parents or guardians, and from patients aged ≥16 years old, with respect to the use of their blood for scientific purposes.

The patient recruitment process under the S168/2018 project took place between March 2019 and January 2022 (a total of 94 patients were included in the study). In the period from October 2023 to February 2024, an additional 29 patients diagnosed at the Children’s Memorial Health Institute (Warsaw, Poland) and the University Children’s Hospital in Lublin (Poland) were included.

### 2.2. ALEX^®^2 Test

The ALEX^®^2 test (Macro Array Diagnostics GmbH, Vienna, Austria) is a third-generation multiplex test for measuring the levels of sIgE against allergen extracts and molecular components simultaneously. ALEX^®^2 helps determine sIgE levels against 295 allergens, including 117 extracts and 178 allergen molecules from various sources, such as foods, animals, plants, and molds. The tested extracts and molecules were classified into 3 allergen groups: inhalant, food, and other (with the last group comprising allergens whose route of exposure is other than via inhalation or ingestion, for instance, via insect stings or via direct dermal contact, such as *Malassezia sympodialis*). The results were exported from MADx Raptor software (version V1.13) to Excel spreadsheets. The results obtained with these tests were quantitative and were expressed in kilounits of sIgE per liter (kU_A_/L). In accordance with the normal sIgE ranges provided by the manufacturer, a test was considered positive if its result was ≥0.3 kU_A_/L.

### 2.3. Statistical Analysis

Statistical analysis was performed using IBM^®^ SPSS^®^ 24.0.0.0. Statistics (Nowy Jork, NY, USA). Relationships between qualitative variables were tested with Spearman’s rank coefficient. The Mann–Whitney U test was used to test for differences in the number of positive results between females and males. The level of statistical significance was set at *p* = 0.05.

## 3. Results

### 3.1. Patients’ Characteristics

Out of 123 patients with CD, 108 children were included in the analysis. Patients were excluded because of a lack of a written informed consent (*n* = 2) or of a proper CD diagnostic procedure (*n* = 13). The study group included 63% girls and 37% boys; the average age of the children was 9.4 years and ranged from 0.9 to 17.4 years. Out of all evaluated patients with CD, the diagnostic protocol without duodenal biopsy was followed in nearly 78% of cases. IgA deficit was detected in 2.8% of study subjects (Table 1). Patients with CD exhibited predominantly gastrointestinal manifestations: abdominal pain (*n* = 39; 36.1%), abdominal distension (*n* = 37; 34.3%), diarrhea (*n* = 32; 29.6%), and constipation (*n* = 6; 5.6%). Other manifestations included weight loss (*n* = 21; 19.4%) and growth retardation (*n* = 10; 9.3%), with more details listed in Table 1.

### 3.2. Sensitization Profile

**Figure 1:**
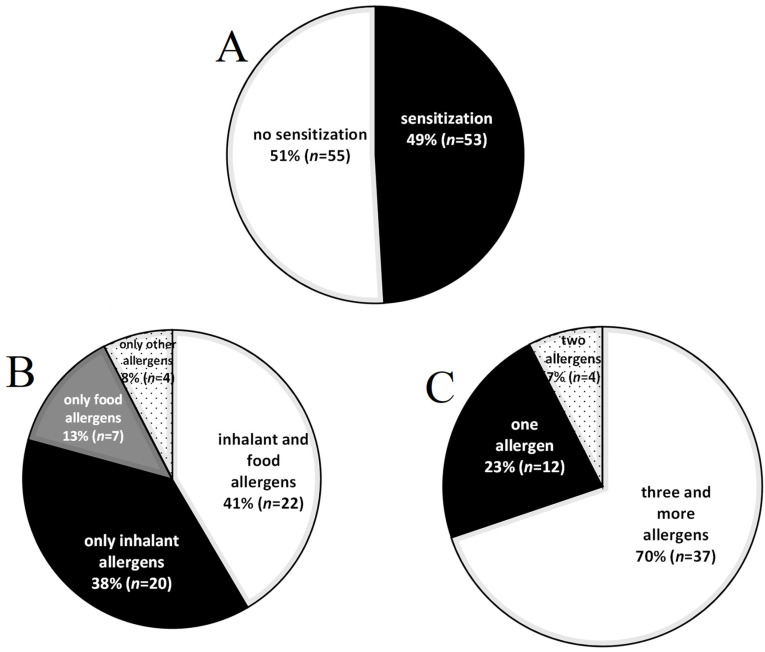
Prevalence of sensitization to allergen extracts and molecules in children with coeliac disease (*n* = 108). (**A**) The percentage of sensitized (black area) and not sensitized (white area) children. (**B**) The percentage of children sensitized only to food (grey area), inhalant (black area), inhalant and food (black area), or other (dotted area) allergens. (**C**) The percentage of children sensitized to one (black area), two (dotted area), or more (white area) allergens. Sensitization was assessed in blood sera of children using the ALEX^®^2 test with the cut-off point of ≥0.3 kU_A_/L for a positive result.

Multiplex tests revealed sIgEs to allergen extracts and/or molecules in 53 out of 108 (53/108) children with CD (49.1%). These children showed sensitization to 145 (44 extracts and 101 molecules) out of all 295 extracts and molecules assessed with the multiplex test. A large subgroup of children with CD (22/53; 41.5%) showed simultaneous sensitivity to food and inhalant allergens (Figure 1). A majority of children (37/53; 69.8%) showed sensitivity to three or more allergens, whereas sIgEs to a single allergen (extract and/or molecule) were observed in twelve (22.6%) and to two or more allergens in four children (7.5%).

Inhalant allergens induced production of the highest concentrations of sIgEs, with the highest levels to black alder (46.71 kU_A_/L) and timothy grass (44.35 kU_A_/L) (Poaceae family) molecules and to *Dermatophagoides farinae* (42.99 kU_A_/L) dust mite (NPC2 family) molecules. The highest sIgE levels to food allergens were produced by exposure to salmon (β-parvalbumin), muskmelon (profilin), and apple (PR-10) molecules (12.38 kU_A_/L, 11.26 kU_A_/L, and 9.09 kU_A_/L, respectively). The 10 most sensitizing allergens in children with CD included 7 inhalants and 2 food allergens. Table 2 shows a list and ranking of the common sensitizing allergens in children with CD.

The presence of sIgEs to inhalant allergens (extracts and molecules) were detected in 38.8% of children with CD (48/108). Most of those children (37/42; 88.1%) showed sensitization to more than one inhalant allergen. Analysis of all extracts and molecules demonstrated the most common sIgEs to be against timothy (26.9%), perennial ryegrass (24.1%), and silver birch (18.5%) pollens (Table 2). These three most common inhalant allergens are allergen molecules Phl p 1, Lol p 1 (both from the beta-expansin subfamily), and Bet v 1 (PR-10 subfamily). Over one-half of the children with CD who showed sensitization to inhalant allergens (22/42; 52.4%) had a coexisting sensitization to food allergens. Sensitization to food allergens (extracts and molecules) was detected in over one-quarter of the study group (29/108; 26.8%), and three-quarters of children (22/29; 75.9%) showed sIgEs against more than one food allergen. The most common food allergen was hazelnut (15/108; 13.95%), which placed as low as eighth in terms of prevalence among all the analyzed allergens listed in Table 2. Somewhat lower in the list of sensitizing agents were apple and peanut allergens (with these allergens placing twelfth and sixteenth, respectively; Table 2). These three highest-placing food allergens were molecules (Cor a 1, Mal d 1, Ara h 8) from the PR-10 subfamily. Over three-quarters of the children with CD and sensitization to food allergens (22/29; 75.9%) showed co-sensitization to inhalant allergens.

Evaluated children had no sIgEs against 150 out of all 295 allergens (73 extracts and 77 molecules) tested, among which were allergens of all gluten-containing cereals, including wheat (*Triticum aestivum*) molecules (Tri a aA_TI (alpha-amylase trypsin inhibitor), Tri a 14 (nsLTP), and Tri a 19 (Omega-5-gliadin)), as well as spelt (*Triticum spelta*), cultivated rye (*Secale cereale*), barley (*Hordeum vulgare*), and oat (*Avena sativa*) extracts.

### 3.3. Relationship between Sensitization and Patients’ Age and Sex

Statistical analysis showed no significant differences in sensitization rates between girls and boys with CD and sensitivity to any extracts or molecules, whether food, inhalant, or other allergens (*p* = 0.462). Moreover, there were no significant differences between boys and girls in the rates of sensitization to food (extracts and molecules) (*p* = 0.674) or inhalant (extracts and molecules) allergens (*p* = 0.665).

Spearman’s rank correlation analysis (Table 3) in sensitized patients showed a significant correlation only between the patient’s age and the occurrence of positive sIgEs (≥0.3 kU_A_/L) to inhalant allergen molecules (*p* = 0.045), which means that the rate of sensitization to inhalant allergen molecules increased with age. There was, however, no significant correlation between the patient’s age and sensitization to food allergens, either extracts or molecules (Table 3).

## 4. Discussion

A systematic review of studies assessing co-existence of IgE-mediated allergy/sensitization and CD [6] revealed two original articles that ruled out [15,16] and only four original articles that suggested the possibility of allergy and CD co-occurrence [8,9,10,11], with a vast majority of authors assessing the co-occurrence of CD and sensitization to food allergens. Our study was conducted in a group of pediatric patients with CD with the use of multiplex tests to assess the levels of serum sIgE against 295 allergens, including 117 extracts and 178 allergen molecules from various sources, such as foods, animals, plants, and molds. To the authors’ knowledge, this is the only analysis assessing such a wide panel of allergens, both extracts and molecules. Assessments conducted in 108 children with CD showed IgE-mediated sensitization in nearly half of the study group (53/108; 49.1%). Other authors reported considerably lower sensitization rates in patients with CD [8,11], which may be related to the methodology they adopted to assess sensitization. Cudowska et al. demonstrated IgE-mediated allergy/sensitization in just over 20% (12/59; 20.3%) of children with CD [11]. Ciacci et al., who evaluated patients older than 17 years newly diagnosed with CD based on questionnaire-reported allergy symptoms (*n* = 1044), their relatives (*n* = 2752), and spouses (*n* = 318), showed that 16.6% of patients with CD were allergic to at least one of the evaluated allergens [8]. The higher sensitization rates shown in our study group with CD may be due to the number and type of assessed allergens. The analysis by Cudowska et al. was based solely on sIgEs against 20 food and inhalant allergens [11]. Similarly, Ciacci et al. assessed sensitization to 20 inhalant and food allergens and conducted skin tests in selected patients [8]. Additionally, unlike previous studies, our study assessed sIgEs against both allergen extracts and molecules, which may increase screening test sensitivity [17,18].

Our study showed children with CD to have the highest rates of sensitization to inhalant allergens. The three allergens with the highest sensitization rates in children with CD were timothy grass (Phl p 1), perennial ryegrass (Lol p 1), and silver birch (Bet v 1) pollens. Cudowska et al. [11], who evaluated the relationship between sensitization to inhalant allergen extracts, showed a similar tendency: the allergens with the second and third highest sensitization rates were grass and birch pollens. However, the highest rates of sensitization in that study were shown to be due to dust mites, whereas in our study, the same dust mite allergen (*Dermatophagoides pteronyssinus* molecule Der p 23) placed seventh in the ranking of the most common sources of inhalation allergies.

The food allergen with the highest sensitization rates in children with CD was hazelnut (Cor a 1), followed by apple (Mal d 1), and peanut (Ara h 8). These allergens placed eighth, twelfth, and sixteenth, respectively, among all the food and inhalant allergen extracts and molecules presented in Table 2. Peanut allergens were also among the most common food allergens in a study by Cudowska et al. [11], who demonstrated that nearly half of the evaluated children showing sensitization to any allergen (5/12; 41.7%) were sensitized to peanuts. However, that analysis was based solely on allergen extracts, and it is difficult to draw conclusions as to this having been a primary peanut sensitization or a result of secondary reaction (cross-reactivity with inhalant allergens).

In our study the three food allergens that placed highest in the ranking presenting in Table 2 (Cor a 1, Mal d 1, and Ara h 8) belong to the PR-10 subfamily, which has been demonstrated to produce cross-reactivity, with primary sensitivity to birch allergens (specifically Bet v 1 of the PR-10 subfamily) [19,20]. Our previous research shows that cases of sensitization to food allergens from plant sources may result from a primary allergy to inhalant allergens [21]. Therefore, it is worth noting that in over three-quarters of the current study group of children with CD and food allergen sensitization showed a co-existing sensitization to inhalant allergens. A reverse situation, namely, food allergen sensitization in children with CD and sensitivity to inhalant allergens, was less common, though a large proportion (over half) of the children with sensitivity to food allergens was still also sensitive to inhalant allergens. Nonetheless, the greatest proportion of children with CD (41.5%) was simultaneously sensitive to inhalant and food allergens. Most children with CD demonstrated sensitivity to two or more allergens, whereas less than one-quarter of the study group was sensitive to a single allergen. Importantly, the same allergen molecules (Cor a 1.0401, Mal d 1, and Ara h 8) were the most common allergens in another of our study, where we assessed the levels of sIgE against food allergen extracts and molecules in 3715 Polish children being diagnosed for a suspected allergy (the mean age of the study population was 7.0 years) [22], which may suggest that the food allergen sensitization profile in patients with CD is similar to that observed in the general Polish population.

In this current study, in children with CD, we observed no sIgEs against 150 (73 extracts or 77 molecules) out of all 295 allergens assessed with the multiplex test. Interestingly, the tested allergens included all gluten-containing cereal allergens: wheat (*Triticum aestivum*) molecules (Tri a aA_TI, alpha-amylase trypsin inhibitor; Tri a 14, nsLTP; and Tri a 19, omega-5-gliadin) as well as spelt (*Triticum spelta*), cultivated rye (*Secale cereale*), barley (*Hordeum vulgare),* and oat (*Avena sativa*) extracts. Studies investigating the co-existence of CD and sensitivity to wheat or other cereals have been inconclusive. Using skin prick and sIgE tests, Armentia et al. [9] demonstrated that 7.0% out of 57 patients (adults and children) with CD were sensitive to wheat [8]. A study conducted by Lanzarin et al. [10] in 74 patients with CD (aged 1–20 years) showed the rates of sensitization to wheat, rye, barley, and barley malt to be 4%, 10.8%, 5.4%, and 2.7%, respectively. Our results are consistent with those reported by other authors, who indicated no sensitization to gluten-containing cereals in patients with CD [11,16]. Interestingly, just like our research team, Spoerl et al. [16], who assessed allergy to wheat extract and wheat molecule Tri a 19, showed no sensitization to the evaluated allergens in patients with CD.

The incidence of CD is higher in women than in men, a fact which has been corroborated in an epidemiological study by Makharia et al. [23], where the pooled incidence rates in women and men were 17.4 (95% CI: 13.7–21.1) and 7.8 (95% CI: 6.3–9.2) per 100,000 person-years, respectively. This trend was also observed in our study group, whose majority (63%) were females. Studies regarding the incidence of sensitization with respect to patient’s sex suggest a relationship with the patient’s age and hormone levels [24]. Boys may be susceptible to allergies at an earlier age than girls, a phenomenon that may be absent later in life. In our study, a statistical analysis of those children with CD who had any sensitivity (to extracts and/or molecules of food, inhalant, or other allergens) showed no significant differences between girls and boys in the incidence of sensitization. The sensitization profile in children changes with age, and the rates of sensitization to inhalant allergens increased with patient age [25]. The observations from the current study also present a significant relationship between the age of children with CD and the number of positive sIgE test results against inhalant allergen molecules. Other studies have also shown this phenomenon in normal populations [26,27].

Due to the fact that in our study, the vast majority of patients (78%) had high concentrations of anti-tTG antibodies, we did not assess the correlation between the concentration of these antibodies and the sensitization profile. It seems that such an analysis on another group of CD patients could demonstrate whether anti-tTG concentration is related to the incidence and type of sensitization.

To assess the clinical forms of allergy, all sensitized patients were invited to the allergy outpatient clinic to undergo a clinical examination. However, so far, only seven patients have returned for their visit. This situation is due to the way the allergy outpatient clinic operates and the feasibility of accommodating all children with CD included in this study. Nonetheless, preliminary data indicate that all children who already had their visit at the clinic reported gastrointestinal symptoms (including abdominal pain, bloating, diarrhea/constipation) or exhibited inadequate weight gain. An interesting observation is that some of these patients presented oral allergy syndromes, probably as a cross-reaction with inhalant allergens, and none of them had a diagnosed food allergy to wheat proteins prior to the diagnosis of CD. These children most commonly reported inhalation allergy and were diagnosed with hay fever, conjunctivitis, or bronchial asthma. Other reported allergy symptoms were skin changes in the form of hives or atopic dermatitis. We are planning to conduct a detailed analysis of the clinical forms of allergy in children with CD.

In addition, further comparisons of the obtained results to the general population are necessary to show whether there is a difference in the sensitization profile between children with CD and their peers without CD.

## 5. Conclusions

Our analysis showed that nearly half of the children with CD were sensitized to at least one allergen, with no cases of sensitivity to gluten-containing cereals. Moreover, children with CD were usually sensitized simultaneously to both inhalant and food allergens. The food allergens with the highest sensitization rates were molecules of the PR-10 subfamily (Cor a 1, Mal d 1, and Ara h 8), which may be due to cross-reactivity with birch, in which its primary allergy marker, Bet v 1 (PR-10 subfamily), was among the three most common inhalant allergens after timothy grass and perennial ryegrass (Phl p 1, Lol p1). In conclusion, the current study indicates a need for IgE-mediated allergy diagnostics in patients with CD, in terms of not only food allergy but also inhalant allergy.

The current study highlights the need to put more emphasis on the diagnosis of IgE-mediated allergy in patients with CD, which may contribute to better patient care and lead to a better understanding of the relationship between CD and IgE-mediated allergy.

## Figures and Tables

**Table 1:** Characteristics of patients with newly diagnosed coeliac disease (CD) included for the analysis of specific IgE prevalence against allergen extracts and molecules.

		Number of Patients	%
Sex	Female	68	63%
	Male	40	37%
Age (in years)	Mean	9.4	
	Standard deviation	4.43	
	Median	9.3	
	Minimum	0.9	
	Maximum	17.4	
CD diagnosis ^1^	Without biopsy	84	78%
	With biopsy	24	22%
IgA deficiency	Yes	3	3%
	No	105	97%
Symptoms ^2^	Abdominal pain	39	36.1%
	Diarrhea	32	29.6%
	Abdominal distension	37	34.3%
	Constipation	6	5.6%
	Growth retardation	15	9.3%
	Weight loss	21	19.4%
	Anemia	15	13.9%
	Skin changes	13	12.0%
	Other ^3^	19	17.6%

^1^ CD was diagnosed according to European Society for Paediatric Gastroenterology Hepatology and Nutrition guidelines [13]; ^2^ multiple symptoms have been observed in individual patients; ^3^ other symptoms include headaches, chronic fatigue, hypersensitivity and hypertransaminasemia.

**Table 2:** List of the most common allergens sensitizing children with coeliac disease, based on the analysis of specific IgEs (sIgEs) to food and inhalant allergen extracts and molecules.

	Allergen	Molecule or Extract	Route of Exposure	Family of Molecules	Number of Patients (%)	Mean sIgE Levels (in kU_A_/L)
1	Timothy grass	Phl p 1	Inhalation	Beta-expansin	29 (26.9%)	18.08
2	Perennial ryegrass	Lol p 1	Inhalation	Beta-expansin	26 (24.1%)	16.03
3	Silver birch	Bet v 1	Inhalation	PR-10	20 (18.5%)	18.20
4	Bermuda grass	Extract	Inhalation		18 (16.7%)	3.96
5	Bermuda grass	Cyn d 1	Inhalation	Beta-expansin	18 (16.7%)	7.02
6	Beech	Fag s 1	Inhalation	PR-10	16 (14.8%)	12.26
7	Timothy grass	Phl p 2	Inhalation	Expansin	16 (14.8%)	16.39
8	Hazelnut	Cor a 1.0401	Ingestion	PR-10	15 (13.9%)	7.57
9	Hazel	Cor a 1.0103	Inhalation	PR-10	15 (13.9%)	13.36
10	*Dermatophagoides pteronyssinus*	Der p 23	Inhalation	Chitinase class III	14 (13.0%)	15.18
11	Timothy grass	Phl p 5.0101	Inhalation	Group 5/6 grass	14 (13.0%)	24.82
12	Apple	Mal d 1	Ingestion	PR-10	13 (12.0%)	9.09
13	Black alder	Aln g 1	Inhalation	PR-10	13 (12.0%)	6.71
14	Hazel	Extract	Inhalation		12 (11.1%)	6.77
15	Rye pollen	Extract	Inhalation		12 (11.1%)	7.33
16	Peanut	Ara h 8	Ingestion	PR-10	12 (11.1%)	3.32
17	Strawberry	Fra a 1+3	Ingestion	PR-10+LTP	12 (11.1%)	7.01
18	Timothy grass	Phl p 6	Inhalation	Group 5/7 grass	11 (10.2%)	23.06
19	*Dermatophagoides farinae*	Der f 2	Inhalation	NPC2 family	10 (9.3%)	42.99
20	*Dermatophagoides pteronyssinus*	Der p 2	Inhalation	NPC2 family	10 (9.3%)	42.14
21	Dog urine (including Can f 5)	Extract	Inhalation		9 (8.3%)	8.46
22	*Paspalum notatum*	Extract	Inhalation		9 (8.3%)	2.23
23	Cat	Fel d 1	Inhalation	Secretoglobin	9 (8.3%)	21.52
24	*Glycyphagus domesticus*	Gly d 2	Inhalation	NPC2 family	9 (8.3%)	7.47
25	Celery	Api g 1	Ingestion	PR-10	8 (7.4%)	6.93
26	Carrot	Dau c 1	Ingestion	PR-10	8 (7.4%)	6.75
27	Soy	Gly m 4	Ingestion	PR-10	8 (7.4%)	2.81
28	*Dermatophagoides farinae*	Der f 1	Inhalation	Cysteine protease	8 (7.4%)	21.92
29	Carrot	Extract	Ingestion		7 (6.5%)	3.35
30	European ash	Extract	Inhalation		7 (6.5%)	9.69
31	*Dermatophagoides pteronyssinus*	Der p 1	Inhalation	Cysteine protease	7 (6.5%)	22.38
32	Olive	Ole e 1	Inhalation	Ole e 1 family	7 (6.5%)	11.10
33	Walnut pollen	Extract	Inhalation		6 (5.6%)	2.74
34	Melon	Cuc m 2	Ingestion	Profilin	6 (5.6%)	11.26
35	European ash	Fra e 1	Inhalation	Ole e 1 family	6 (5.6%)	8.47
36	Annual mercury	Mer a 1	Inhalation	Profilin	6 (5.6%)	3.13
37	Date palm	Pho d 2	Inhalation	Profilin	6 (5.6%)	12.26
38	Common wasp venom	Ves v 5	Other	Antigen 5	6 (5.6%)	1.91

sIgEs were assessed in blood sera with an ALEX^®^2 test. The cut-off point for a positive result was ≥0.3 kU_A_/L. The first column presents the ranking of frequency of sIgEs to allergens in the study group; 1 reflects the highest frequency, while 38 reflects the lowest one.

**Table 3:** Association between the age of children with coeliac disease (CD) and the prevalence of sensitivities to inhalant and food allergen extracts and molecules, diagnosed based on serum sIgE levels.

	Number of Sensitized Children	*r*	*p*-Value
All tested allergens	53	0.21	0.124
Food extracts and molecules	29	0.13	0.491
Inhalant extracts and molecules	42	0.30	0.055
Food extracts	20	0.20	0.403
Food molecules	23	0.18	0.411
Inhalant extracts	28	0.27	0.161
Inhalant molecules	42	0.31	0.045 ^1^

^1^ A statistically significant result (*p* < 0.05). The relationship between age of children with CD and the prevalence of sensitivities to allergen extracts and molecules was analyzed with Spearman’s rank correlation test; *r* = Spearman’s rank coefficient. Sensitization was assessed in blood sera of children using the ALEX^®^2 test with the cut-off point of ≥0.3 kU_A_/L for a positive result.

## Data Availability

The data presented in this study are available on request from the corresponding authors (B.C. and E.M.). The data are not publicly available due to ethic restrictions.
